# Association Between Right Ventricular Dysfunction and Coronary Angiographic Complexity in Patients With Acute Myocardial Infarction

**DOI:** 10.7759/cureus.109086

**Published:** 2026-05-18

**Authors:** Kazi Mohammad Faisal, Asish Dey, Md Nur Uddin Tareq, Md Enamul Hoque, Md Shahidullah, Md Mofazzol Hoque, Md Mahadi Hassan, Sakir Hossain, Mohammad Abdul Mannan, Tahmina Akhter

**Affiliations:** 1 Department of Cardiology, Chittagong Medical College Hospital, Chattogram, BGD; 2 Department of Cardiology, Shaheed Tajuddin Ahmad Medical College Hospital, Gazipur, BGD; 3 Department of Cardiology, Chattagram International Medical College and Hospital, Chattogram, BGD; 4 Department of Cardiology, Directorate General of Health Services (DGHS), Dhaka, BGD; 5 Department of Cardiology, Faujderhat Chest Disease Hospital, Chattogram, BGD; 6 Department of Cardiology, Fatikchari Upazila Health Complex, Chattogram, BGD; 7 Department of Cardiology, Khulna Medical College Hospital, Khulna, BGD; 8 Department of Dermatology, Chittagong Medical College Hospital, Chattogram, BGD

**Keywords:** acute myocardial infarction, bangladesh, coronary artery disease, echocardiography, right ventricular dysfunction, syntax score

## Abstract

Background and aim

Acute myocardial infarction (AMI) remains a major cause of cardiovascular morbidity and mortality worldwide, particularly in low- and middle-income countries. Although right ventricular dysfunction (RVD) has emerged as an important determinant of outcomes in AMI, its relationship with coronary angiographic complexity remains inadequately explored. This study aimed to evaluate the association between RVD and coronary angiographic complexity in patients with AMI.

Methods

A hospital-based, prospective observational study was conducted at Chittagong Medical College Hospital, Bangladesh, from March 2024 to February 2025. A total of 98 patients with AMI were included via consecutive sampling. Right ventricular function was assessed using echocardiographic parameters, including tricuspid annular plane systolic excursion (TAPSE), S′ velocity, and fractional area change (FAC). Coronary angiographic complexity was evaluated using the SYNTAX score. The data were analyzed using IBM SPSS Statistics for Windows, version 23.0 (released 2015; IBM Corp., Armonk, NY, USA). Appropriate comparative tests and multivariable logistic regression analysis were performed to explore independent predictors while considering the limited number of outcome events.

Results

The mean age of the participants was 54.9 ± 11.5 years, and 85.71% were male. RVD was significantly associated with intermediate and high SYNTAX scores (p = 0.002). Among patients with RVD, 47.0% had low SYNTAX scores, whereas 26.5% and 26.5% had intermediate and high SYNTAX scores, respectively. In contrast, among patients without RVD, 76.9% had low SYNTAX scores, whereas 14.3% and 6.1% had intermediate and high scores, respectively. Patients with RVD had significantly lower systolic blood pressure (120.9 ± 18.3 vs 132.6 ± 23.5 mmHg, p = 0.013). Echocardiographic parameters, including TAPSE (16.3 ± 4.2 vs 19.2 ± 4.5 mm, p = 0.002) and FAC (25.6 ± 10.7% vs 35.9 ± 11.3%, p < 0.001), were significantly reduced in patients in the intermediate and high SYNTAX score groups. In the multivariable analysis, RVD showed the strongest association with intermediate and high SYNTAX scores (OR = 7.86, 95% CI: 2.53-24.39, p < 0.001), followed by diabetes mellitus (OR = 5.44, 95% CI: 1.78-16.68, p = 0.003).

Conclusions

RVD was significantly associated with greater coronary angiographic complexity in patients with AMI. It was more common among patients with intermediate and high SYNTAX scores and remained independently associated with increased angiographic complexity. These findings suggest that RVD may serve as a useful noninvasive marker for early risk stratification. Larger multicenter studies are needed to confirm its prognostic significance.

## Introduction

Acute myocardial infarction (AMI) continues to be a major contributor to cardiovascular morbidity and mortality worldwide. Recent estimates suggest that its occurrence increases with age, affecting approximately 3.8% of individuals under 60 years of age and nearly 9.5% of those aged 60 years or older, indicating an increasing burden with increasing age [1]. Globally, more than three million people develop ST-elevation myocardial infarction (STEMI) each year, highlighting the scale of acute coronary events [2]. Data from the Global Burden of Disease 2021 study revealed that ischemic heart disease, with AMI as its most severe form, accounted for approximately 9.0 million deaths and 188.4 million disability-adjusted life years worldwide [3]. Despite improvements in reperfusion therapy and preventive measures, AMI remains a significant cause of cardiovascular mortality, especially in low- and middle-income countries (LMICs) [4].

Coronary angiography is the standard method for assessing the anatomical extent and complexity of coronary artery disease (CAD) in patients with AMI. The SYNTAX score is a validated angiographic tool that incorporates lesion number, location, and morphological features to determine disease complexity and assist in guiding revascularization strategies [5]. Its prognostic value is well recognized, as higher SYNTAX scores are linked to increased rates of major adverse cardiovascular events (MACE), cardiac death, and repeat revascularization [6]. Moreover, progressive increases in the SYNTAX score have been associated with poorer clinical outcomes, particularly in patients with complex coronary lesions such as left main disease [7].

The right ventricle (RV), previously considered functionally passive, is now recognized as a critical determinant of outcomes in AMI. Right ventricular dysfunction (RVD) may occur due to direct ischemic injury, most commonly from proximal right coronary artery occlusion, or indirectly through interventricular dependence and elevated left-sided filling pressures. Clinical evidence has demonstrated that patients with RVD experience significantly higher rates of adverse cardiovascular events and mortality than those with preserved RV function [8]. In large observational cohorts, RVD has been independently associated with increased in-hospital mortality and reduced long-term survival, particularly among patients with cardiogenic shock complicating AMI [9].

Echocardiography is a noninvasive method for assessing RV function. Key parameters such as tricuspid annular plane systolic excursion (TAPSE), tissue Doppler-derived systolic velocity (S′), and right ventricular fractional area change (FAC) are recommended by current guidelines as reliable indicators of RV systolic performance [10]. These indices are easily obtainable and cost-effective and have demonstrated good correlation with cardiac magnetic resonance-derived measurements, making them practical tools for routine clinical assessment and risk stratification [11].

Emerging evidence suggests a potential link between RV dysfunction and the complexity of underlying CAD. Studies have shown that extensive CAD, particularly multivessel involvement, may impair RV function through compromised myocardial perfusion and reduced coronary reserve [12]. In addition, RVD has been identified as a strong predictor of adverse cardiovascular outcomes in patients with CAD, including those undergoing surgical revascularization [13]. However, despite these observations, data directly correlating echocardiographic RV dysfunction with angiographic complexity as quantified by the SYNTAX score remain limited.

This issue is particularly relevant in LMICs, where the burden of AMI is increasing while access to advanced diagnostic and interventional facilities remains restricted. In these settings, coronary angiography is often not immediately available, and patients frequently present late with advanced disease, contributing to poorer outcomes [14,15]. Bangladesh exemplifies this scenario, with cardiovascular diseases accounting for nearly one-third of all deaths and a high prevalence of modifiable risk factors such as smoking, hypertension, and diabetes mellitus [16,17].

Despite the recognized prognostic importance of RVD in AMI, its relationship with angiographic disease complexity has not been adequately explored in the Bangladeshi population. Identifying a simple, noninvasive marker such as RVD that correlates with coronary complexity could have important implications for early risk stratification and clinical decision-making, particularly in resource-limited settings. Therefore, this study aimed to evaluate the association between RVD and coronary angiographic complexity in patients with AMI.

## Materials and methods

Study design and setting

A hospital-based, prospective observational study was conducted in the Department of Cardiology, Chittagong Medical College Hospital (CMCH), Chattogram, Bangladesh. The study was carried out over a one-year period from March 2024 to February 2025. CMCH is a tertiary care referral center providing specialized cardiac services, including coronary angiography and echocardiography.

Study population

The study population comprised adult patients diagnosed with AMI, including both STEMI and non-STEMI (NSTEMI), who were admitted to the Department of Cardiology during the study period. The diagnosis of AMI was established on the basis of clinical presentation, electrocardiographic findings, and relevant biochemical markers. Patients who underwent coronary angiography during index hospitalization were considered eligible. Individuals with unstable angina, pericardial disease, cardiomyopathy, chronic lung disease, pulmonary hypertension, connective tissue disorders, moderate-to-severe valvular heart disease, or atrial fibrillation were excluded. Patients unwilling to provide informed consent were also excluded.

Sampling procedure and sample size

A consecutive sampling technique was used to recruit participants. Eligible patients were enrolled consecutively after applying predefined inclusion and exclusion criteria and providing written informed consent. The sample size was calculated using the following formula:

$n = \frac{(Z_{\alpha} + Z_{\beta})^{2} \, (\sigma_{1}^{2} + \sigma_{2}^{2})}{(\mu_{1} - \mu_{2})^{2}}$

Using a 95% confidence level (Zα = 1.96), 80% power (Zβ = 0.842), mean SYNTAX scores of 22.4 and 18.8, and SDs of 5.2 and 7.3, respectively, from a previous study by Keskin et al., the required sample size was estimated to be 49 patients in each group [18]. Accordingly, a minimum sample size of 98 participants was required. Eligible patients were enrolled consecutively during the study period. After data collection, the participants were categorized into two groups on the basis of the presence or absence of RVD for comparative analysis.

Data collection procedures

Data were gathered using a structured case record form. Following informed consent, detailed clinical history, physical examination findings, and relevant laboratory investigations were documented. Transthoracic echocardiography was performed within 24 hours of admission using a standardized echocardiography machine (Affiniti 30, Philips Healthcare, Andover, MA, USA) by an experienced cardiologist who was blinded to both the clinical and electrocardiographic findings.

Assessment of right ventricular function

Right ventricular function was assessed using TAPSE, tricuspid annular S′, and FAC. RVD was defined when at least two of the following parameters were abnormal: FAC <35%, TAPSE <17 mm, and S′ <10 cm/s [10]. Coronary angiography was performed during index hospitalization via a transradial approach using a dedicated angiography system (Shimadzu Trinias, Shimadzu Corporation, Kyoto, Japan). For most patients, coronary angiography was performed within the same hospitalization period after stabilization following the index AMI event according to institutional practice and clinical condition. Angiographic images were evaluated by an expert cardiologist blinded to the echocardiographic findings.

Assessment of coronary angiographic complexity (SYNTAX score)

Coronary angiographic complexity was assessed using the SYNTAX score, a validated tool for quantifying the complexity and extent of CAD. All coronary angiograms were analyzed by an experienced interventional cardiologist who was blinded to the echocardiographic findings. The SYNTAX score was calculated using the standard online SYNTAX scoring algorithm [19]. Each coronary lesion producing ≥50% diameter stenosis in vessels ≥1.5 mm in diameter was identified and scored individually. Lesions were evaluated on the basis of multiple anatomical and morphological characteristics, including lesion location, total occlusion, bifurcation or trifurcation involvement, severe tortuosity, heavy calcification, thrombus presence, and lesion length. The individual lesion scores were summed to obtain the total SYNTAX score for each patient. Patients were initially categorized into three groups according to the total SYNTAX score: low (≤22), intermediate (23-32), and high (>32) to describe the distribution of angiographic complexity. For further analytical purposes, including logistic regression, the intermediate and high categories were combined into a single group (>22) to represent intermediate and high SYNTAX score groups [20].

Statistical analysis

The data were reviewed for completeness and subsequently analyzed using IBM SPSS Statistics for Windows, version 23.0 (released 2015; IBM Corp., Armonk, NY, USA). Continuous variables are presented as mean ± SD for normally distributed data, whereas categorical variables are summarized as frequencies and percentages. The distribution of continuous variables was assessed using the Shapiro-Wilk test. Comparisons between patients with and without RVD, as well as between low and intermediate/high SYNTAX score groups, were performed using the independent-samples t-test or Mann-Whitney U test for continuous variables and the chi-square test or Fisher’s exact test for categorical variables, as applicable. Spearman’s rank correlation coefficient was used to evaluate associations between right ventricular functional parameters and SYNTAX score. Variables found to be significant in bivariate analysis were included in a multivariable binary logistic regression model to determine predictors of intermediate and high SYNTAX score groups. ORs with 95% CIs were calculated, and a p-value <0.05 was considered statistically significant.

Ethical considerations

Ethical approval was obtained from the Ethical Review Committee of Chittagong Medical College (approval 59.27.0000.013.19.PG.009.2024/1064). The study was conducted in accordance with the principles of the Declaration of Helsinki. Written informed consent was obtained from all participants prior to enrollment. Confidentiality and anonymity were strictly maintained, and participants were informed of their right to withdraw at any stage without any consequences.

## Results

The mean age of the participants was 54.9 ± 11.5 years. The majority of the patients were male, accounting for 84 (85.71%), whereas females accounted for 14 (14.29%). A high prevalence of cardiovascular risk factors was observed among the study population. Hypertension was present in 78 (79.59%) patients, followed by current or recent smoking in 73 (74.49%) patients. Diabetes mellitus was identified in 35 (35.71%) patients, dyslipidemia in 16 (16.33%), and obesity in 18 (18.37%). A positive family history of ischemic heart disease was noted in only 7 (7.14%) patients. Regarding clinical presentation, an equal number of patients presented with STEMI and NSTEMI, each accounting for 49 (50.00%) patients (Table 1).

**Table 1:** Baseline sociodemographic and clinical characteristics of the study population (n = 98) AMI, acute myocardial infarction; IHD, ischemic heart disease; NSTEMI, non-ST-elevation myocardial infarction; STEMI, ST-elevation myocardial infarction

Variable	Category	Frequency (n)	Percentage (%)
Age	Mean ± SD	54.9 ± 11.5	
Sex	Male	84	85.71
	Female	14	14.29
Smoking status	Current/recent	73	74.49
	No	25	25.51
Hypertension	Yes	78	79.59
	No	20	20.41
Diabetes mellitus	Yes	35	35.71
	No	63	64.29
Dyslipidemia	Yes	16	16.33
	No	82	83.67
Obesity	Yes	18	18.37
	No	80	81.63
Family history of IHD	Yes	7	7.14
	No	91	92.86
AMI type	STEMI	49	50.00
	NSTEMI	49	50.00

A comparison of clinical and laboratory parameters between patients with and without RVD is presented in Table 2. The two groups were comparable in terms of age (54.3 ± 11.3 vs 55.5 ± 11.7 years, p = 0.605), heart rate (85.8 ± 10.7 vs 86.2 ± 11.1 beats/min, p = 0.846), fasting blood sugar level (159.6 ± 59.1 vs 158.2 ± 64.9 mg/dL, p = 0.912), and serum creatinine level (1.1 ± 0.3 vs 1.2 ± 0.3 mg/dL, p = 0.781). However, systolic blood pressure was significantly lower among patients with RVD than among those without RVD (120.9 ± 18.3 vs 132.6 ± 23.5 mmHg, p = 0.013). No statistically significant difference was observed in diastolic blood pressure between the two groups (74.1 ± 9.9 vs 74.9 ± 8.4 mmHg, p = 0.913).

**Table 2:** Comparison of clinical and laboratory parameters between patients with and without RVD DBP, diastolic blood pressure; FBS, fasting blood sugar; RVD, right ventricular dysfunction; SBP, systolic blood pressure

Variable	RVD (n = 49), mean ± SD	No RVD (n = 49), mean ± SD	p-Value
Age (years)	54.3 ± 11.3	55.5 ± 11.7	0.605
Heart rate (/min)	85.8 ± 10.7	86.2 ± 11.1	0.846
SBP (mmHg)	120.9 ± 18.3	132.6 ± 23.5	0.013
DBP (mmHg)	74.1 ± 9.9	74.9 ± 8.4	0.913
FBS (mg/dL)	159.6 ± 59.1	158.2 ± 64.9	0.912
Serum creatinine (mg/dL)	1.1 ± 0.3	1.2 ± 0.3	0.781

Echocardiographic parameters differed significantly between patients with and without RVD, as shown in Table 3. Patients with RVD had markedly reduced TAPSE (14.4 ± 2.2 mm vs 21.9 ± 3.1 mm, p < 0.001), S′ velocity (9.6 ± 1.8 cm/s vs 13.9 ± 2.4 cm/s, p < 0.001), and FAC (21.9 ± 6.8% vs 42.2 ± 6.3%, p < 0.001).

**Table 3:** Comparison of echocardiographic parameters according to RVD status FAC, fractional area change; RVD, right ventricular dysfunction; TAPSE, tricuspid annular plane systolic excursion

Variable	RVD	No RVD	p-Value
TAPSE (mm)	14.4 ± 2.2	21.9 ± 3.1	<0.001
Tricuspid annular velocity S′ (cm/s)	9.6 ± 1.8	13.9 ± 2.4	<0.001
FAC, %	21.9 ± 6.8	42.2 ± 6.3	<0.001

Angiographic findings are presented in Table 4. Thrombi were observed in 12 (25.0%) patients in the RVD group, whereas none (0.0%) of the patients in the non-RVD group had thrombi (p < 0.001). Chronic total occlusion (CTO) was also significantly more common in the RVD group than in the non-RVD group (28 (57.1%) vs 11 (22.4%), p < 0.001). Other angiographic features, including multivessel disease (41 (83.7%) vs 34 (69.4%), p = 0.095), left main disease (10 (20.4%) vs 5 (10.2%), p = 0.161), and heavy calcification (10 (20.4%) vs 4 (8.2%), p = 0.083), were more common among patients with RVD, although these differences were not statistically significant.

**Table 4:** Comparison of angiographic characteristics between patients with and without RVD CTO, chronic total occlusion; RVD, right ventricular dysfunction

Variable	RVD (n = 49)	No RVD (n = 49)	p-Value
Multivessel disease	41 (83.7)	34 (69.4)	0.095
Left main disease	10 (20.4)	5 (10.2)	0.161
Heavy calcification	10 (20.4)	4 (8.2)	0.083
Thrombus	12 (25.0)	0 (0.0)	<0.001
CTO	28 (57.1)	11 (22.4)	<0.001

The distribution of angiographic complexity based on the SYNTAX score according to RVD status is shown in Table 5. A significantly greater angiographic burden was observed among patients with RVD than among those without RVD (p = 0.002). Among patients with RVD, 23 (47.0%) had low SYNTAX scores, whereas 13 (26.5%) and 13 (26.5%) had intermediate and high scores, respectively. In contrast, among patients without RVD, the majority, 39 (76.9%), had low SYNTAX scores, whereas only 7 (14.3%) and 3 (6.1%) had intermediate and high scores, respectively.

**Table 5:** Distribution of angiographic complexity on the basis of SYNTAX score according to RVD status RVD, right ventricular dysfunction

Variable	RVD	No RVD	p-Value
Low (≤22)	23 (47.0)	39 (76.9)	0.002
Intermediate (23-32)	13 (26.5)	7 (14.3)	
High (>32)	13 (26.5)	3 (6.1)	

The associations of baseline variables with angiographic complexity are presented in Table 6. Diabetes mellitus was significantly more common among patients with intermediate/high SYNTAX scores than among those with low SYNTAX scores (18 (50.0%) vs 17 (27.4%), p = 0.025). Hemodynamic parameters also showed significant associations. Patients in the intermediate and high SYNTAX score groups had lower systolic blood pressure (113.1 ± 24.1 vs 124.1 ± 19.9 mmHg, p = 0.016) and diastolic blood pressure (71.8 ± 10.0 vs 76.9 ± 8.1 mmHg, p = 0.008). Echocardiographic parameters were strongly associated with angiographic complexity. Patients with intermediate/high SYNTAX scores had lower TAPSE (16.3 ± 4.2 vs 19.2 ± 4.5 mm, p = 0.002) and FAC (25.6 ± 10.7% vs 35.9 ± 11.3%, p < 0.001). RVD was significantly more prevalent among patients in the intermediate and high SYNTAX score groups (26 (72.2%) patients) than in the low SYNTAX score group (23 (37.1%) patients) (p = 0.001).

**Table 6:** Comparison of baseline characteristics between patients with low and intermediate/high SYNTAX scores AMI, acute myocardial infarction; DBP, diastolic blood pressure; FAC, fractional area change; IHD, ischemic heart disease; NSTEMI, non-ST-elevation myocardial infarction; RVD, right ventricular dysfunction; SBP, systolic blood pressure; SS, SYNTAX score; STEMI, ST-elevation myocardial infarction; TAPSE, tricuspid annular plane systolic excursion

Variable	Category	Low SS (n = 62)	Intermediate/high SS (n = 36)	p-Value
Age (years)	Mean ± SD	54.3 ± 11.9	55.9 ± 10.5	0.497
Sex	Male	54 (87.1)	30 (83.3)	0.608
	Female	8 (12.9)	6 (16.7)	
Smoking	Current/recent	45 (72.6)	28 (77.8)	0.569
	No	17 (27.4)	8 (22.2)	
Hypertension	Yes	50 (80.6)	28 (77.8)	0.734
	No	12 (19.4)	8 (22.2)	
Diabetes mellitus	Yes	17 (27.4)	18 (50.0)	0.025
	No	45 (72.6)	18 (50.0)	
Dyslipidemia	Yes	9 (14.8)	7 (19.4)	0.548
	No	53 (85.2)	29 (80.6)	
Obesity	Yes	11 (17.7)	7 (19.4)	0.834
	No	51 (82.3)	29 (80.6)	
Family history of IHD	Yes	2 (3.2)	5 (13.9)	0.096
	No	60 (96.8)	31 (86.1)	
Heart rate (/min)	Mean ± SD	85.6 ± 9.7	86.8 ± 12.7	0.631
SBP (mmHg)	Mean ± SD	124.1 ± 19.9	113.1 ± 24.1	0.016
DBP (mmHg)	Mean ± SD	76.9 ± 8.1	71.8 ± 10.0	0.008
FBS (mg/dL)	Mean ± SD	155.6 ± 50.2	160.8 ± 67.9	0.074
Serum creatinine (mg/dL)	Mean ± SD	1.2 ± 0.3	1.1 ± 0.3	0.692
AMI type	STEMI	37 (59.7)	15 (41.7)	0.085
	NSTEMI	25 (40.3)	21 (58.3)	
TAPSE (mm)	Mean ± SD	19.2 ± 4.5	16.3 ± 4.2	0.002
S′ velocity (cm/s)	Mean ± SD	12.2 ± 2.9	10.9 ± 3.2	0.051
FAC (%)	Mean ± SD	35.9 ± 11.3	25.6 ± 10.7	<0.001
RVD	Present	23 (37.1)	26 (72.2)	0.001
	Absent	39 (62.9)	10 (27.8)	

The results of the multivariable logistic regression analysis for intermediate and high SYNTAX scores are presented in Table 7. In this model, diabetes mellitus and RVD remained significantly associated with intermediate and high SYNTAX scores. Patients with diabetes mellitus had more than fivefold higher odds of having intermediate or high SYNTAX scores (OR = 5.44, 95% CI: 1.78-16.68; p = 0.003). RVD showed the strongest association, with nearly eightfold higher odds of intermediate or high SYNTAX scores (OR = 7.86, 95% CI: 2.53-24.39; p < 0.001). Other included variables did not show statistically significant associations in the multivariable model.

**Table 7:** Multivariable logistic regression analysis for intermediate and high SYNTAX scores DBP, diastolic blood pressure; NSTEMI, non-ST-elevation myocardial infarction; RVD, right ventricular dysfunction; SBP, systolic blood pressure; STEMI, ST-elevation myocardial infarction

Variable	Category	β	OR	95% CI	p-Value
Diabetes mellitus	Present vs absent	1.695	5.44	1.78-16.68	0.003
Family history of IHD	Present vs absent	0.813	2.25	0.34-15.05	0.401
SBP (mmHg)	Per unit increase	-0.014	0.986	0.960-1.013	0.296
DBP (mmHg)	Per unit increase	-0.059	0.943	0.882-1.007	0.081
AMI type	STEMI vs NSTEMI	0.485	1.62	0.60-4.43	0.344
RVD	Present vs absent	2.062	7.86	2.53-24.39	<0.001

## Discussion

In the present study, we aimed to evaluate the association between RVD and coronary angiographic complexity in patients with AMI. Our findings demonstrated that RVD was significantly associated with greater angiographic complexity and a higher prevalence of intermediate/high SYNTAX scores. RVD also remained significantly associated with intermediate and high SYNTAX scores, whereas echocardiographic parameters were strongly associated with angiographic complexity.

One of the principal findings of the present study was the significant association between RVD and greater coronary angiographic complexity. This association appears biologically plausible and may be explained through several pathophysiological mechanisms. The RV receives its primary blood supply from the right coronary artery, and proximal right coronary artery occlusion is a well-recognized cause of RV ischemia in AMI [21]. However, RVD in AMI may not be attributable solely to isolated right coronary artery involvement. In patients with extensive CAD, impaired left ventricular (LV) systolic function can adversely affect RV performance through ventricular interdependence, with a substantial proportion of RV systolic function depending on LV contractility [22]. Moreover, RV dilatation within the relatively noncompliant pericardial space may increase intrapericardial pressure, reduce LV filling, and impair coronary perfusion, thereby contributing to progressive biventricular dysfunction, particularly in the setting of multivessel disease [23]. These mechanisms are supported by previous clinical studies demonstrating that RVD is associated with increased mortality and poorer long-term outcomes in AMI patients, especially among those with cardiogenic shock [9]. In addition, a previous single-center study involving STEMI patients reported significantly higher rates of MACE among patients with reduced TAPSE, further suggesting that impaired RV function may reflect a greater underlying burden and complexity of CAD [8].

Another important observation was the strong and graded association between reduced echocardiographic parameters, namely TAPSE, S′ velocity, and FAC, and intermediate and high SYNTAX score groups. These indices represent longitudinal and global RV systolic function, and their impairment may reflect both direct ischemic injury to the RV myocardium and indirect effects of LV dysfunction in the presence of extensive coronary disease [24]. Previous studies have consistently demonstrated the prognostic value of these parameters in AMI. In a prospective cohort of STEMI patients, reduced TAPSE, FAC, and S′ velocity were independently associated with increased in-hospital complications and reduced survival [25]. The present findings are therefore consistent with existing evidence and support the use of routine echocardiographic assessment of RV function as a surrogate marker of coronary disease complexity.

The study also demonstrated a significant association between diabetes mellitus and increased angiographic complexity. Patients with diabetes had substantially greater odds of having intermediate or high SYNTAX scores, which is consistent with the findings of previous studies. Diabetes is known to accelerate atherosclerosis through endothelial dysfunction, oxidative stress, chronic inflammation, and platelet activation, leading to diffuse and complex coronary lesions [26]. A clinical study has shown that poor glycemic control is associated with higher SYNTAX scores and more extensive coronary involvement [27], and diabetes has been identified as an independent predictor of CAD complexity in multivariable analyses [28]. The observed association between lower blood pressure and intermediate and high SYNTAX scores in this study may reflect hemodynamic compromise due to advanced coronary disease rather than a direct causal relationship.

With respect to angiographic characteristics, patients with RVD had a significantly greater incidence of coronary thrombus and CTO. These findings further support the association between RVD and severe CAD. CTO is a marker of longstanding and diffuse atherosclerosis and has been independently associated with increased mortality in patients with STEMI [29]. A large cohort study demonstrated that the presence of a non-culprit CTO is linked to significantly increased short- and long-term mortality [30]. Similarly, the presence of intracoronary thrombus reflects plaque instability and acute coronary occlusion, indicating high-risk coronary anatomy. Taken together, the increased prevalence of CTO and thrombus in patients with RVD suggests that impaired RV function may serve as an indicator of a more extensive and hemodynamically significant coronary disease burden.

This study is among the first from Bangladesh to evaluate the association between RVD and coronary angiographic complexity using the SYNTAX score in patients with AMI. Standardized and blinded echocardiographic and angiographic assessments helped reduce measurement bias. However, several limitations should be considered. The single-center design, relatively small sample size, and observational nature of the study may limit generalizability and preclude causal inference. Long-term prognostic outcomes were not evaluated. Culprit vessel-specific analysis was not performed, limiting assessment of whether RVD was related to specific coronary anatomy or overall coronary disease burden. Residual confounding from factors such as infarct location, reperfusion status, and medication exposure could not be fully excluded. In addition, the limited number of outcome events may have introduced a risk of model overfitting; therefore, the regression findings should be interpreted cautiously and considered exploratory.

## Conclusions

RVD was significantly associated with greater coronary angiographic complexity in patients with AMI and was more frequently observed among patients with intermediate and high SYNTAX scores. In the multivariable analysis, RVD remained significantly associated with greater angiographic complexity, whereas echocardiographic parameters such as TAPSE and FAC also demonstrated significant relationships with SYNTAX score complexity. These findings suggest that impaired right ventricular function may serve as a useful noninvasive marker for early risk stratification and may reflect a greater underlying burden of CAD. Nevertheless, larger multicenter studies with longitudinal follow-up and more comprehensive angiographic assessment are needed to further validate these findings and clarify their prognostic implications.
